# Lymphocyte CD4/CD8 ratio predicts early immune-related toxicity in lung cancer patients treated with immune checkpoint inhibitors: a single-center retrospective study

**DOI:** 10.3389/fmed.2026.1829572

**Published:** 2026-06-08

**Authors:** Wenfang Jin, Ying Zhao, Yanling Lv, Yu Yao, Xin Su

**Affiliations:** 1 Department of Respiratory and Critical Care Medicine, The Second Hospital of Nanjing, Nanjing University of Chinese Medicine, Nanjing, China; 2 Department of Respiratory and Critical Care Medicine, Xuzhou TCM Hospital Affiliated to Nanjing University of Chinese Medicine, Xuzhou, China; 3 Department of Respiratory and Critical Care Medicine, Nanjing Drum Tower Hospital, The Affiliated Hospital of Nanjing University Medical School, Nanjing, China

**Keywords:** biomarker, CD4/CD8 ratio, immune checkpoint inhibitor, immune-related adverse events, lung cancer

## Abstract

**Background:**

We determined whether the baseline peripheral blood CD4/CD8 ratio predicts grade ≥2 immune-related adverse events (irAEs) within 30 days of initiating single-agent PD-1/PD-L1 blockade in advanced lung cancer.

**Method:**

In this single-center retrospective study (January 2022–December 2025), we included adults with pathologically confirmed advanced NSCLC who received first-line treatment with pembrolizumab, tislelizumab, sintilimab, and camrelizumab. Baseline flow cytometry performed within 7 days before cycle 1 was required. The primary endpoint was grade ≥2 irAEs within 30 days. Multivariate logistic regression was used to identify independent predictors and evaluate model performance (area under the curve [AUC]).

**Results:**

Among 202 patients, 51 (25%) developed grade ≥2 irAEs. Median onset ranged from 2.1 weeks (rash) to 3.0 weeks (endocrine). Baseline CD4/CD8 ratio < 1.65 remained was present in 63% of patients with early irAE vs. 36% of controls (*p* < 0.001). After adjustment, ratio <1.65 remained the predictor (odds ratio [OR] 3.03; 95% confidence interval [CI] 1.57–5.84), while hypertension (OR 4.37) and diabetes (OR 4.08) added modest risk. The final model achieved an AUC of 0.78 (95% CI 0.71–0.85).

**Conclusion:**

A pre-treatment CD4/CD8 ratio <1.65 remained is a simple, inexpensive biomarker that identifies lung cancer patients at high risk for early irAEs during single-agent PD-1/PD-L1 blockade. Prospective, multicenter validation is warranted.

## Introduction

Lung cancer is the leading cause of cancer-related death worldwide (1). Immune checkpoint inhibitors (ICIs) that block the programmed death-1 pathway have become the standard first-line treatment for advanced non-small cell lung cancer (NSCLC) (2). Pembrolizumab approximately doubles median overall survival compared with cytotoxic therapy (3, 4). Real-world data have confirmed similar magnitudes of benefit, although the median age was 5 years older and the performance status distribution was broader than in the pivotal studies (5). In China, domestic PD-1 antibodies (sintilimab and camrelizumab) and the CTLA-4 inhibitor tislelizumab are reimbursed, and the response rate is comparable to that of pembrolizumab (6).

These effective therapies increase the risk of immune-related adverse events (irAEs) (7–10). Pooled analyses show that early grade ≥2 immune-related toxicities are common, with pneumonitis, colitis, and hepatitis accounting for the majority of cases (11–13). Importantly, 70% of grade ≥3 events occur within the first 4 weeks, and each day of delayed steroid treatment increases the probability of intensive care admission by 3% (14). Traditional clinical variables (age, sex, body mass index [BMI], Eastern Cooperative Oncology Group [ECOG] performance status, histology, smoking status, hypertension, and diabetes) have not reliably identified high-risk patients (15, 16). While PD-1/PD-L1 inhibitors are typically combined with chemotherapy for advanced NSCLC, single-agent pembrolizumab, sintilimab, tislelizumab, or camrelizumab represent viable alternatives for elderly (≥75 years) or chemotherapy-ineligible patients, as demonstrated by improved survival and safety in trials such as IPSOS, thereby enabling the investigation of monotherapy toxicity patterns and biomarkers. In melanoma, a baseline CD4/CD8 ratio <1.0 doubled the risk (17). Peripheral blood T-cell profiling offers an alternative. The CD4/CD8 ratio integrates helper and cytotoxic immunity in a single universally available metric. A low ratio signals effector-dominant immunity and has been linked to autoimmune flares in rheumatoid arthritis and to cytokine release syndrome after CAR-T therapy (18, 19). In melanoma, a baseline CD4/CD8 ratio <1.0 doubles the risk of grade ≥3 immune-related adverse events (20). A small lung cancer series observed that 63% of patients who developed early irAEs had a ratio <1.65, compared with 36% of those who did not (21). To our knowledge, no prior study has tested whether this threshold predicts acute toxicity in a real-world Chinese cohort exposed only to single-agent PD-1/ PD-L1 blockade. Existing data are limited to small, mixed chemotherapy cohorts or non-Chinese populations.

We therefore conducted a single-center retrospective study to determine whether a CD4/CD8 ratio <1.65 is associated with grade ≥2 irAEs within the first 30 days of initiation of pembrolizumab, sintilimab, tislelizumab, and camrelizumab monotherapy for advanced lung cancer.

## Methodology

We conducted a retrospective study at the hospital to determine whether the baseline peripheral blood CD4/CD8 ratio predicts irAEs in patients with advanced lung cancer who received single-agent ICIs.

### Patient selection

Patients ≥18 years old with pathologically confirmed advanced NSCLC who were not fit for platinum doublet chemotherapy and received first-line single-agent PD-1 or PD-L1 monotherapy between January 2022 and December 2025 were screened. Inclusion criteria were: the use of a single-agent checkpoint inhibitor was restricted to patients who were not fit for platinum doublet chemotherapy due to age ≥75 years, ECOG performance status ≥2, or any medical contraindications; at least one measurable lesion; baseline flow cytometry performed within 7 days before the first infusion; and complete follow-up data for 30 days. Exclusion criteria: Patients who received any cytotoxic agent, including anti-CTLA-4 antibody or radiotherapy, within 2 weeks of cycle 1; had a prior organ transplant; had active autoimmune disease; had a concurrent malignancy; or received combined checkpoint blockade were excluded.

### Data collection

Demographics, smoking history, ECOG performance status, histology, PD-L1 tumor proportion score, treatment regimen, laboratory results, and irAE records were extracted from the electronic database. irAEs were graded using CTCAE v5.0 by investigators blinded to lymphocyte counts; events recorded beyond 30 days were censored.

### Flow cytometry analysis

A 2 mL sample of peripheral blood was collected in EDTA-K₂ tubes and processed within 4 h on a BD FACS Canto II using the BD Multi-TEST IMK kit. The lymphocytes were identified by forward scatter (FSC) vs. side scatter (SSC) and then followed by CD3 + gating. The CD3 + population was further analyzed for CD4 + and CD8 + T cells using BD Trucount tubes to determine absolute cell counts. FACSDiva software was used to calculate the CD4/CD8 ratio. At least 10,000 events per sample were required. The intra-assay coefficient of variation was <5% for the CD4/CD8 ratio. Systematic inter-run coefficient-of-variation data were not captured in this retrospective study. We adopted a CD4/CD8 cutoff of 1.65 *a priori*, based on previous Chinese cohorts (21).

### Statistical analysis

All analyses were performed using SPSS 26.0. Continuous variables are presented as mean ± standard deviation (SD) and were compared using the independent *t*-test; categorical variables are expressed as *n* (%) and were analyzed with χ^2^ test or Fisher’s exact test. Two-sided *p*-values of <0.05 were considered statistically significant. Multivariate logistic regression included variables with *p*-values <0.10 in univariate analysis.

## Results

A total of 202 patients were included. Mean age was 61.6 ± 10.6 years in the early irAE group and 64.3 ± 9.6 years in the no-irAE group; 68.6% vs. 62.9% were male, 60.8% vs. 48.3% had ECOG performance status 0–1, 84.3% vs. 80.8% had NSCLC histology, and 33.3% vs. 45.0% received pembrolizumab, sintilimab, or camrelizumab with no significant between-group differences (all *p* > 0.05). A baseline CD4/CD8 ratio <1.65 was more frequent in patients who subsequently developed early immune-related adverse events (63% vs. 36%, *p* < 0.001). Early immune-related adverse events occurred in 51 patients, as shown in Table 1.

**Table 1:** Patient demography.

Parameters	Early irAE Yes (*n* = 51)	Early irAE No (*n* = 151)	*P*-value
Age	61.63 ± 10.56	64.32 ± 9.64	0.094
Male sex	35 (68.6%)	95 (62.9%)	0.523
BMI	24.2 ± 3.8	24.3 ± 4.1	0.892
ECOG 0-1 (*n* = 104)	31 (60.8%)	73 (48.3%)	0.073
NSCLC histology (*n* = 202)	51 (25.2%)	151 (74.8%)	
Smoking history	42 (82.4%)	128 (84.8%)	0.700
Current smoker	12 (23.5%)	38 (25.2%)	
Ex-smoker	30 (58.8%)	90 (59.6%)	
Never smoked	9 (17.6%)	23 (15.2%)	
Hypertension	22 (43.1%)	68 (45.0%)	0.818
Diabetes	12 (23.5%)	29 (19.2%)	0.432
Regimen (*n* = 202)			0.452
Pembrolizumab	17 (33.3%)	68 (45.0%)	
Tislelizumab	23 (45.1%)	55 (36.4%)	
Sintilimab	5 (9.8%)	16 (10.6%)	
Camrelizumab	6 (11.8%)	12 (7.9%)	
CD4/CD8 ratio			<0.001
<1.65	32 (62.7%)	54 (35.8%)	
≥1.65	19 (37.3%)	97 (64.2%)	

### Lymphocyte subsets

Patients who developed early irAEs exhibited significantly lower CD4 T-cell counts (585.0 ± 94.5 vs. 747.9 ± 95.7 cells/μL, *p* < 0.001) and a lower CD4/CD8 ratio (1.35 ± 0.16 vs. 1.85 ± 0.27, *p* < 0.001), as shown in Table 2. The baseline CD4/CD8 ratio was 1.84 (IQR 1.65–2.05) in patients without irAEs and 1.34 (IQR 1.26–1.48) in those who developed irAEs. Both distributions were approximately normal. The *a priori* cutoff of 1.65 fell between the two group medians and effectively stratified the cohort, with 63% of irAE cases occurring below this threshold vs. 37% of controls.

**Table 2:** Baseline lymphocytes.

Parameters	Early irAE Yes (*n* = 51)	Early irAE No (*n* = 151)	*P*-value
CD4 T cells (cells/μL)	585.0 ± 94.5	747.9 ± 95.7	<0.001
	585.0 (520–650)	748.0 (680–815)	
CD8 T cells (cells/μL)	433.9 ± 52.2	406.8 ± 43.6	<0.001
	433.9 (390–478)	406.8 (375–440)	
CD4/CD8 ratio	1.35 ± 0.16	1.85 ± 0.27	<0.001
	1.34 (1.26–1.48)	1.84 (1.65–2.05)	

### IrAE profile

Early immune-related adverse events occurred in 51 patients. Rash was most frequent (26 patients; 23 grade 1, 3 grade 2), followed by pneumonitis (10 patients; 2 grade 1, 7 grade 2, 1 grade ≥3), colitis (8 patients; 3 grade 1, 4 grade 2, 1 grade ≥3), and endocrine events (7 patients; all grade 1). No grade ≥3 toxicity was recorded for rash or endocrine irAEs, as shown in Table 3.

**Table 3:** Immune-related adverse events.

IrAE type	Patients	Grade 1	Grade 2	Grade ≥3	% of total irAEs
Rash	26	23	3	0	51.0%
Endocrine	7	7	0	0	13.7%
Colitis	8	3	4	1	15.7%
Pneumonitis	10	2	7	1	19.6%
Total	51	35	14	2	100.0%

Rash appeared earliest (median 2.1 weeks, 1.4–2.9), followed by endocrine events (2.9 weeks, 2.3–3.7), colitis (2.7 weeks, 2.0–3.5), and pneumonitis (3.0 weeks, 2.1–3.9), indicating that the majority of immune-related adverse events occurred within the first 4 weeks of immune checkpoint inhibitor therapy, as shown in Table 4.

**Table 4:** Time to onset of immune-related adverse events.

Immune-related adverse events	Time to onset (weeks)	Patients, *n* (%)	Grade ≥3 *n* (%)
Rash	2.1 (1.4–2.9)	26 (51.0%)	0 (0%)
Endocrine	2.9 (2.3–3.7)	7 (13.7%)	0 (0%)
Colitis	2.7 (2.0–3.5)	8 (15.7%)	1 (12.5%)
Pneumonitis	3.0 (2.1–3.9)	10 (19.6%)	1 (10.0%)

### Multivariate analysis

Baseline CD4/CD8 ratio <1.65 was a strong independent predictor of grade ≥2 irAEs (OR 3.03, 95% CI 1.57–5.84, *p* < 0.001). Hypertension (OR 4.37, 95% CI 1.29–14.74, *p* = 0.018) and diabetes (OR 4.08, 95% CI 1.06–15.74, *p* = 0.041) contributed additional modest risk, as shown in Table 5.

**Table 5:** Multivariate logistic regression predicting irAEs.

Parameter	OR (95% CI)	*P*
CD4/CD8 ratio <1.65	3.03 (1.57–5.84)	<0.001
Age	0.97 (0.92–1.03)	0.325
ECOG **≥**2 (vs. 0–1)	3.78 (0.81–17.61)	0.090
Male sex	0.83 (0.26–2.64)	0.753
Hypertension	4.37 (1.29–14.74)	0.018
Diabetes	4.08 (1.06–15.74)	0.041

### Performance of CD4/CD8 ratio lymphocytes

The performance of the CD4/CD8 ratio as a continuous biomarker was evaluated. We performed ROC analysis using the inverse-transformed ratio (1/ratio) to account for the direction of association. The AUC was 0.951 (95% CI 0.921–0.980, *p* < 0.001), indicating excellent discrimination. The *a priori* cutoff of 1.65 demonstrated a sensitivity of 62.7% and specificity of 64.2%, with a Youden index of 0.27. Comparison with alternative cutoffs showed that 1.0 yielded higher sensitivity (98%) but lower specificity (15%), whereas 2.0 improved specificity (80%) at the cost of sensitivity (45%). The data-derived optimal cutoff (1.61–1.72) was concordant with the *a priori* threshold of 1.65, supporting its validity in this cohort (Table 6).

**Table 6:** Performance of the CD4/CD8 ratio cutoff for predicting early irAEs.

Cutoff	Sensitivity (%)	Specificity (%)	PPV	NPV	Youden Index
1.0	98.0	15.2	28.3	95.8	0.13
1.65	62.7	64.2	37.2	83.4	0.27
2.0	45.1	80.1	39.4	84.2	0.25

### Comparison of lymphocytes predictors

We further assessed whether individual lymphocyte counts or ratios best predicted early irAEs. In Model A, CD4 count was independently protective (OR 0.975, 95% CI 0.967–0.984, *p* < 0.001), whereas CD8 count was a risk factor (OR 1.039, 95% CI 1.023–1.056, *p* < 0.001). Model B (dichotomous ratio <1.65) demonstrated superior interpretability (OR 3.03, 95% CI 1.57–5.84, *p* < 0.001) (Table 7).

**Table 7:** Comparison of lymphocyte predictors for early irAEs.

Model	Predictor	OR (95% CI)	*P*-value
Model A CD4 + CD8 continuous	CD4	0.975 (0.967–0.984)	<0.001
	CD8	1.039 (1.023–1.056)	<0.001
Model B (ratio dichotomous)	CD4/CD8 < 1.65	3.03 (1.57–5.84)	<0.001

## Discussion

This study demonstrates that a simple, universally available baseline biomarker—the peripheral blood CD4/CD8 ratio— robustly predicts the risk of early, clinically relevant immune-related adverse events in patients with advanced lung cancer treated with single-agent PD-1 or PD-L1 inhibitors. We found that 63% of participants with a ratio <1.65 experienced grade ≥2 toxicity within the first 30 days of therapy, whereas only 36% of those with a ratio ≥1.65 did so. After adjustment for age, sex, performance status, and comorbidities, a ratio <1.65 remained a strong independent predictor of early irAEs (OR 3.03, 95% CI 1.57–5.84). To our knowledge, this is one of the first independent validations of the 1.65 threshold in a Chinese cohort restricted to single-agent PD-1/PD-L1 inhibitors. While Huang *et al.* established this cutoff in a mixed population of lung cancer patients (21), our study extends their findings by focusing on monotherapy and early irAEs.

Our findings extend previous observations from melanoma and mixed solid tumor cohorts in three important ways. First, we focus on early-onset irAEs (30 days) rather than toxicity occurring over the entire treatment period, which is clinically important because 70% of grade ≥3 irAEs occur within the first month, and each day of steroid delay increases the probability of ICU by 3%. Second, we restricted the treatment to single-agent ICIs, thereby eliminating confounding from CTLA-4/PD-1 combinations that could prolong immune activation. Third, we enrolled patients receiving four different ICIs, including pembrolizumab, tislelizumab, sintilimab, and camrelizumab, thereby reflecting the full spectrum of reimbursed immunotherapies currently available in China, focusing on early onset irAEs and by restricting treatment to single agent ICIs, thereby eliminating confounding from CTLA-4/PD-1 combinations or prolonged follow-up (14, 22).

The 25% incidence of grade ≥2 irAEs we documented within 30 days is virtually identical to the 26% (95% CI 20–32%) 30-day rate reported in a pooled analysis of pembrolizumab monotherapy (23). This concordance provides independent external validation of our early-onset estimate and suggests that our single-center study is representative of a broader immunotherapy-treated population.

The association between a low CD4/CD8 ratio and early toxicity is biologically plausible. Pre-clinical studies show that individuals with a low ratio harbor PD-1 high, effector-dominant CD8^+^ T cells that recover cytotoxic capacity within days once PD-1 signaling is interrupted. These activated T cells traffic to normal tissues and produce IFN-*γ* and granzyme B, thereby precipitating organ-directed autoimmunity (24, 25). The parallel rise in absolute CD8 counts that we observed in patients who did not develop irAEs supports the concept that a balanced or helper-biased milieu restrains bystander tissue injury (26). Conversely, the significant reduction in absolute CD4 counts observed in the early irAE group suggests that insufficient regulatory helper activity may fail to curb effector expansion, a hypothesis that warrants direct investigation in prospective trials.

In addition to the CD4/CD8 ratio, multivariate analysis identified hypertension and diabetes as modest but independent predictors of early irAEs (odds ratio ≈ 4 each). These findings align with emerging real-world databases showing that metabolic endothelial activation amplifies irAE severity (27–29). Chronic low-grade inflammation in patients with hypertension or diabetes increases endothelial adhesion molecule expression and facilitates T-cell extravasation into normal organs. Once checkpoint inhibition is initiated, this primed endothelium may lower the threshold for clinically apparent tissue injury. Importantly, both comorbidities are routinely captured in oncology clinics, allowing instantaneous risk stratification at no additional cost. Several unmeasured factors may alter the association between the CD4/CD8 ratio and irAE. Even low-dose corticosteroids can reduce the number of circulating lymphocytes and modulate the distribution of T-cell subsets (30). Chronic viral infections, such as HIV and CMV, has been known to deplete CD4 + T cells and decrease the CD4/CD8 ratio (31). We excluded patients with active autoimmune disease or chronic viral infection, but we were unable to systematically capture data on baseline corticosteroid or chronic viral infection in this retrospective cohort. Further prospective studies should consider these variables through standardized screening.

The spectrum of organ toxicity observed in our study showed that rash was the most common (51%), followed by pneumonitis (20%), colitis (16%), and endocrine events (14%). Only two grade 3 events were recorded. Median onset ranged from 2.1 weeks (rash) to 3.0 weeks (endocrine), confirming that most clinically relevant early events cluster within the first 30 days.

Our study has several limitations. First, it is a single-center retrospective study. This may limit generalizability to Western populations. The findings require validation in large, prospective cohorts, as the conservative criteria of 10 events per variable were not met. This increases the risk of overfitting and selection bias when univariate screening is applied. We were also unable to collect serial samples after cycle 1, and therefore could not evaluate dynamic changes in the CD4/CD8 ratio or correlate these changes with steroid initiation. Furthermore, the total number of grade ≥3 events was small, precluding meaningful subgroup analysis of severe toxicity. Nevertheless, the identical 1.65 cut-off and the 63% vs. 36% event split we observed replicated the findings of Huang et al. in an independent Chinese cohort (20), providing robust external validation. Taken together, these data support incorporating the baseline CD4/CD8 ratio into routine immunotherapy protocols as a simple, inexpensive, and reproducible biomarker. Our study cohort reflects the clinical indication for PD-1/PD-L1 monotherapy; therefore, the findings may not apply to patients treated with chemo-immunotherapy combinations. Pending prospective validation, patients identified as high risk could be offered enhanced monitoring, early dermatology or pulmonary review, and prompt steroid administration at the first sign of symptoms. Conversely, low-risk patients might be reassured and spared unnecessary investigations, thereby improving resource allocation and quality of life.

## Conclusion

The baseline CD4/CD8 ratio is a simple, inexpensive, and reproducible biomarker that identifies lung cancer patients at high risk of early irAEs during single-agent PD-1/PD-L1 blockade. Prospective, multicenter validation is warranted before integrating this metric into routine immunotherapy protocols.

## Data Availability

The original contributions presented in the study are included in the article/supplementary material, further inquiries can be directed to the corresponding author.
